# Streptococcus pyogenes Pneumonia: A Rare and Severe Presentation in a Patient With Asthma

**DOI:** 10.7759/cureus.47182

**Published:** 2023-10-17

**Authors:** Aimen Iqbal, Daniya Muhammad Haroon, Sanya Badar, Lavleen Kaur, Muhammad Waqas, Faryal Haider, Muneebuddin Syed, Karim Djekidel

**Affiliations:** 1 Internal Medicine, The Wright Center for Graduate Medical Education, Scranton, USA; 2 Internal Medicine, Bahria University Medical and Dental College, Karachi, PAK; 3 Internal Medicine, Bhaskar Medical College, Yenkapally, IND; 4 Critical Care, Geisinger Commonwealth School of Medicine, Scranton, USA

**Keywords:** infection, streptococcus pyogenes, influenza, empyema, pneumonia

## Abstract

Pneumonia is a common respiratory infection typically caused by pathogens such as *Streptococcus pneumoniae*, *Haemophilus influenzae*, and *Staphylococcus aureus*. It is characterized by inflammation and infection in the lung parenchyma, often presenting with symptoms such as cough, fever, and difficulty breathing. Empyema, on the other hand, is a severe complication of pneumonia marked by the accumulation of pus in the pleural cavity. *Streptococcus pyogenes *(*S. pyogenes*), also known as group A *Streptococcus* (GAS), is a bacterium that can cause various infections, including pharyngitis and skin infections. In rare cases, it can lead to community-acquired pneumonia. In our case report, we describe a 32-year-old female with a history of mild persistent asthma who contracted influenza B virus, eventually developing pneumonia caused by GAS, *S. pyogenes*.

## Introduction

*Streptococcus pyogenes* (*S. pyogenes*) is a major human pathogen that causes a wide range of infections, from minor to severe and invasive diseases with major morbidity and mortality. The M protein (emm type) plays an important role in pathogenicity [1]. Typically, it causes pharyngeal and skin infections. *S. pyogenes* is a relatively uncommon cause of community-acquired pneumonia [2]. Diabetes, hypertension, and respiratory disorders (chronic obstructive pulmonary disease (COPD), asthma, and so on) are frequently documented in instances of *S. pyogenes* pneumonia. Acute viral respiratory tract infection, including influenza, is followed by pneumonia caused by *S. pyogenes* [3]. Our case report involves a patient with a history of asthma who was infected with the influenza B virus, which led to pneumonia with a rare bacterium, *S. pyogenes*.

## Case presentation

**Figure 1:**
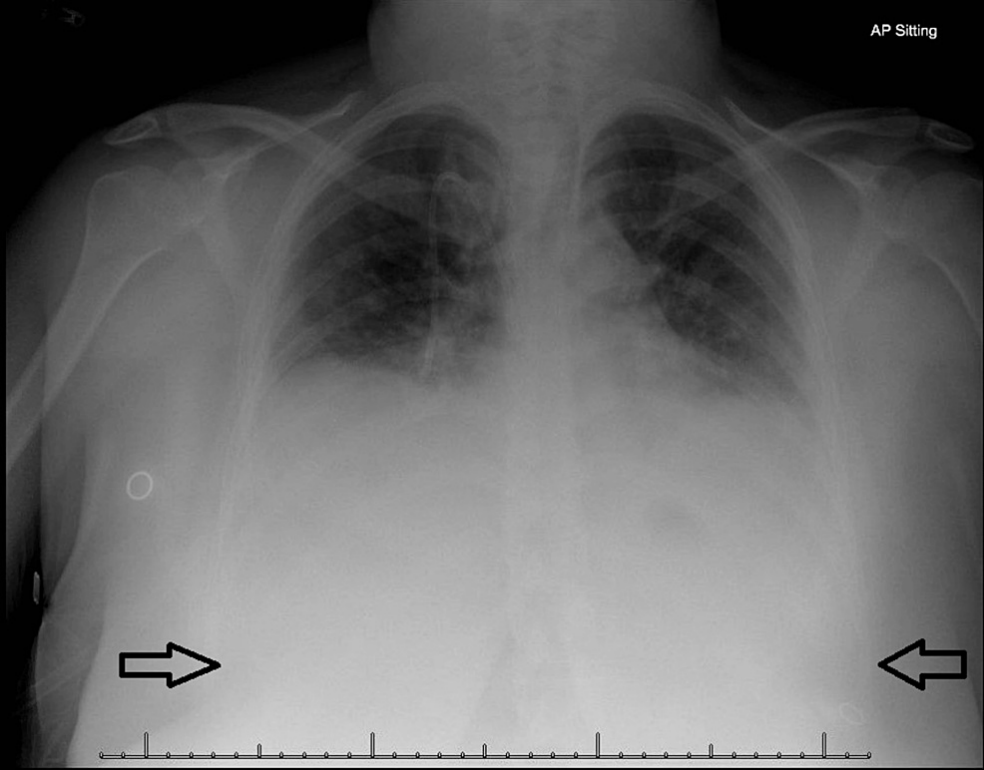
Chest radiograph with the arrows depicting moderate bilateral pleural effusions.

Our patient was a 32-year-old female who had a past medical history significant for mild persistent asthma. She had a 4.5-pack-year smoking history with a quit date 12 years before the presentation. She presented to the emergency department (ED) with complaints of myalgias, chills, nausea, and vomiting. She was diagnosed with influenza B virus infection by a respiratory pathogen panel in the ED and was discharged with advice for symptomatic therapy with acetaminophen and ibuprofen. After four days, she presented to the ED again with worsening shortness of breath and productive cough with blood-tinged sputum. She was hemodynamically unstable on presentation with a blood pressure of 70/48 mmHg, pulse rate of 103 beats/minute, respiratory rate of 25 breaths/minute, and temperature of 97.5°F. Her oxygen saturation was 91%. A physical examination revealed crackles in bilateral lungs and sternal retractions. Laboratory tests showed a high white blood cell (WBC) count of 18.47 x 10^9^ cells/L (normal: 4.00-10.80 x 10^9^ cells/L), lactate of 4.2 mmol/L (normal: 0.4-2.0 mmol/L), blood urea nitrogen concentration of 43 mg/dL (normal: 6-20 mg/dL), and procalcitonin of >100.0 ng/mL (normal: <0.10 ng/mL). All of these variables were above the normal range. Arterial blood gas showed a pH of 7.16 (normal: 7.35-7.45), pCO_2_ of 61.9 mmHg (normal: 35-45 mmHg), pO_2_ of 79.0 mmHg (normal: 75-100 mmHg) and HCO_3_ of 21 mmol/L (normal: 21-32 mmol/L). Urinalysis was positive for protein (>500), esterase (trace), WBC (10-19), WBC clumps, and red blood cells (30-49). Urine *Legionella *and *Streptococcus pneumoniae* antigens were negative. A chest radiograph showed moderate bilateral pleural effusions (Figure 1).

**Figure 2:**
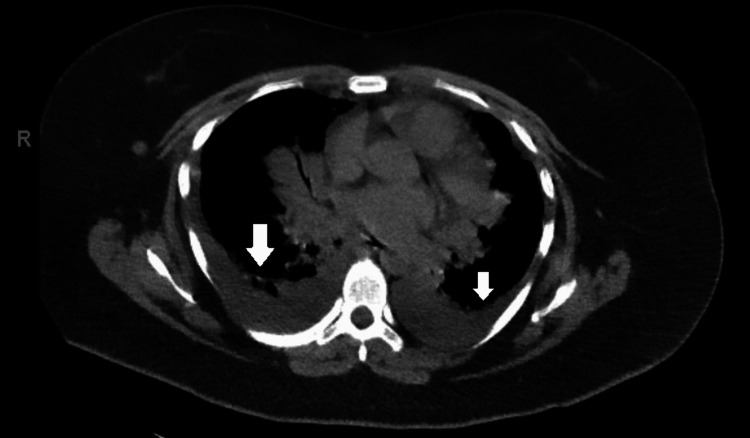
Computed tomography of the chest without contrast with arrows depicting small bilateral pleural effusions.

**Figure 3:**
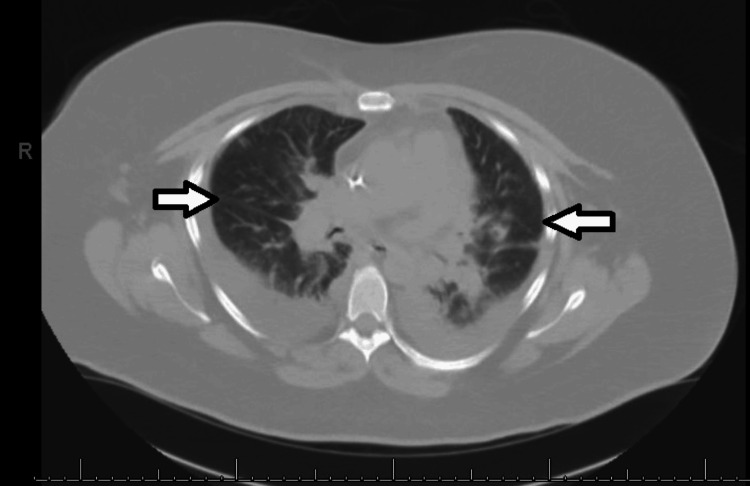
Computed tomography scan demonstrating patchy ground-glass opacities in bilateral lower lobes.

Computed tomography (CT) of the chest without contrast confirmed the moderate bilateral pleural effusions (Figure 2). It also revealed patchy ground-glass opacities in bilateral lower lobes suggestive of consolidations (Figure 3).

Echocardiography showed no abnormalities. Sputum culture and blood culture samples were collected at the time of admission.

**Figure 4:**
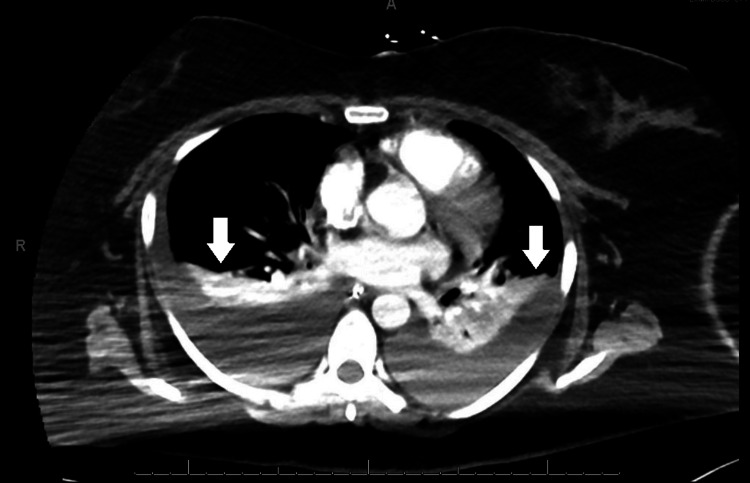
Computed tomography angiogram with arrows depicting enlarging bilateral pleural effusions.

The patient was in obvious respiratory distress at the time of admission; hence, a trial of bi-level positive airway pressure (BiPAP) was done in the ED. She did not tolerate BiPAP and oxygen supplementation with a high-flow nasal cannula was attempted. She was initially saturating well on 50% fraction of inspired oxygen (FiO_2_); however, following the CT scan, she developed increased work of breathing and restlessness as well as worsening hypoxia requiring up-titration of FiO_2_. Given her restlessness, prior intolerance to BiPAP, hypercarbia on venous blood gas (pH -> 7.14 pCO_2_ -> 65.4 pO_2_ -> 43.0), and altered mentation, intubation was favored. She was admitted to the intensive care unit (ICU) with the diagnosis of acute hypercarbic respiratory failure secondary to acute exacerbation of asthma due to influenza infection. Intravenous administration of cefepime (1 g/day) and vancomycin was started along with oral administration of azithromycin 500 mg once a day for broad-spectrum coverage. She also received nebulization with bronchodilators. Following admission, a sputum smear detected no acid-fast bacteria and blood cultures showed no growth. Notably, beta-hemolytic group A streptococci were detected in sputum culture and were identified as *S. pyogenes*. Due to the methicillin-resistant Staphylococcus aureus test being negative, vancomycin was discontinued. As cultures did not show evidence of gram-negative rods, cefepime was discontinued and intravenous ceftriaxone 2 g/day was started, as directed by Infectious Disease specialists. Despite antibiotic therapy, the patient’s oxygenation worsened, indicated by a falling PaO_2_/FiO_2_ ratio. Ventilator settings were optimized in accordance with the acute respiratory distress protocol and the patient was paralyzed and proned. Reevaluation with a CT angiogram performed on day eight revealed enlarging bilateral pleural effusions (Figure 4). She received intravenous methylprednisolone (starting at 60 mg four times daily that was tapered down to 40 mg daily) for 11 days which was then transitioned to oral prednisone (40 mg daily tapered to 5 mg daily) for 10 days.

Bilateral thoracentesis was performed and samples were sent for analysis (Table 1).

**Table 1:** Bilateral thoracentesis findings.

	Right	Left
Clarity	Turbid	Turbid
Total nucleated cell count (cells/µL)	63,160	66,100
Neutrophils (%)	84	74
pH	6.95	7.28
Protein (g/dL)	4.3	4.0
Albumin (g/dL)	2.0	2.0
Glucose (mg/dL)	81	72
Lactate dehydrogenase (U/L)	>2,500 U/L	>2,500 U/L
Pleural fluid gram stain	No growth	Gram-positive cocci

**Figure 5:**
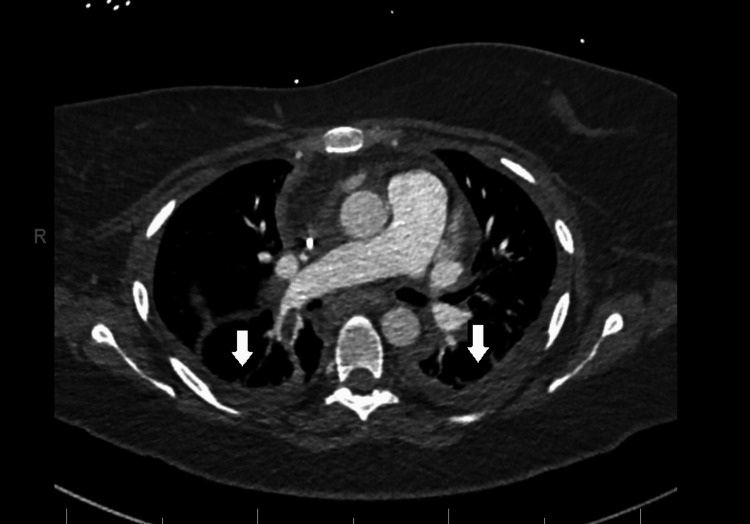
Computed tomography imaging of the chest with arrows depicting reduced bilateral effusions.

Based on Light’s criteria, the pleural fluid protein/serum protein ratio was 0.70 on the right (>0.50 = exudate) and 0.67 on the left. The patient received tissue plasminogen activator (t-PA) and DNase in both chest tubes. The dose of DNase was 5 mg, and the dose of t-PA was 10 mg. They were given intrapleurally twice daily for three days, followed by clamping of the drain to permit the drugs to stay in the pleural space for one hour. Following this therapy, imaging showed reduced bilateral effusions (Figure 5). The patient was also started on intravenous clindamycin 900 mg every eight hours due to the development of empyema.

Following prolonged mechanical ventilation, the patient was successfully liberated from the ventilator on day 13. The patient was hemodynamically stable and was able to tolerate oral intake; hence, intravenous antibiotics were discontinued. Antibiotics were switched to oral amoxicillin/clavulanate for a 14-day course. The left chest tube was removed on day 20, and the right chest tube was removed on day 24. She was discharged on day 34 on 3 L of oxygen via a nasal cannula. On outpatient follow-up two weeks post-discharge, she was noted to remain stable on the same oxygen requirements.

## Discussion

Pneumonia is commonly caused by *Streptococcus pneumoniae*, *Haemophilus influenzae*, *Staphylococcus *spp. (particularly *S. aureus*), and other *Streptococcus *spp. [4,5]. Our case is unique and describes the rare presentation of influenza-superimposed bacterial lung infection caused by *S. pyogenes*. Without any significant trend throughout the course of the study period, there were between one and eight occurrences per year (annual average incidence: 1.14 episodes per 100,000 people; range: 0.29 to 2.29) [3].

Group A *Streptococcus *(GAS) can colonize humans asymptomatically or usually cause pharynx and skin infections. However, *S. pyogenes*, as a rare cause of community-acquired pneumonia, may exhibit a high mortality rate [2], respond slowly to antibiotics, and lead to complications with a prolonged course [6]. Barnham et al. [2] reviewed 17 cases of *S. pyogenes* pneumonia and reported a high mortality rate (47%). *S. pyogenes* pneumonia usually occurs in winter and spring. Common comorbidities in patients with *S. pyogenes* pneumonia include diabetes, hypertension, and COPD. Pleural effusion, cavity formation in the lungs, septicemia, and septic shock are reported complications of *S. pyogenes* pneumonia. Sakai et al. documented a case of invasive GAS infection in an otherwise healthy adult male. This infection initially presented as community-acquired pneumonia and subsequently led to the development of pleural empyema. Ultimately, the patient experienced streptococcal toxic shock syndrome as a severe complication of this infection [7]. Symptoms include a high fever and chest pain, with possible confusion and meningeal signs. Rapid pleural effusion accumulation in patients with acute respiratory infection, observed in up to 80% of *S. pyogenes* pneumonia cases, is the most common symptom suggesting this diagnosis, as seen in our patient. In rare cases, symptoms similar to scarlet fever, such as a desquamating rash, may be noted [7]. The time from the onset of *S. pyogenes* pneumonia to hospitalization can range from one to nine days, with a mean duration of 4.8 ± 2.4 days [8]. In our patient, the duration was four days from onset to hospitalization.

In previous similar case reports on *S. pyogenes* pneumonia, patients had a history of respiratory illnesses (such as asthma, COPD, and pulmonary tuberculosis) and showed co-infection of influenza B virus and *S. pyogenes*. Acute viral tract infections have been associated with *S. pyogenes* pneumonia due to virus-induced epithelial damage, altered or decreased ciliary activity of the respiratory epithelium, which favors bacterial infection, and transient immune suppression [4]. Many bacterial virulence factors are expressed by GAS strains, including protein M, streptolysins, and streptokinase. Protein M, expressed by the *EMM *gene and resistant to phagocytosis, is a significant bacterial virulence factor. Among 82 invasive GAS isolates in Japan, the *EMM1 *gene was the most common (32.9%) and was substantially associated with poor outcomes [1]. During the research period, there was an increase in the incidence of invasive *S. pyogenes* illness, with M1 and M3 strains being the most commonly isolated and linked with pneumonia and deep soft tissue infections [9]. The influenza virus induces an immune response. During invasive pneumococcal superinfection, Barthelemy et al. documented that influenza virus-induced IL-10 production reduced the antibacterial activity of invariant natural killer T cells [10]. According to Robinson et al., the influenza virus suppresses bacteria-induced IL-1 production and weakens human defense against bacterial infection [11]. These cytokines produced by the influenza virus and regulatory T cells may be involved in the immunological mechanisms enabling influenza virus-GAS superinfection. Secondary bacterial superinfection has been observed to develop one week after influenza infection [12,13]. In our patient, respiratory symptoms occurred four days after influenza infection. Tamayo et al. have suggested that influenza B virus could exacerbate *S. pyogenes* pneumonia [3]. As a result, physicians should exercise caution in patients with *S. pyogenes* pneumonia who have a history of influenza virus infection. GAS appears to cause higher morbidity and a longer hospital stay compared to *S. pneumonia* patients. It is more commonly isolated from pleural fluid in the absence of bacteremia, prompting a pleural fluid tap for appropriate diagnosis.

Influenza vaccination can lower the risk of secondary bacterial infections. Studies have shown a decrease in *S. pyogenes* infections in people who had received an influenza vaccination [14,15]. Immediate antibiotic therapy is essential.

In June 2022, Mainali et al. reported a case of a 57-year-old female with a large right exudative effusion complicated with parapneumonic effusion treated with t-PA and DNAse therapy leading to the formation of hemothorax on the first treatment with t-PA [16]. On the other hand, our patient showed a positive response to treatment to t-PA and DNAse. In October 2020, Cheong et al. documented cases involving individuals who presented with complex pleural effusion and experienced suboptimal drainage through intercostal chest catheters. Two of these patients were within the advanced age group, aged over 80 years, making them less suitable candidates for surgical interventions due to the associated risks. However, the research findings demonstrated the successful treatment of all patients through a minimally invasive approach, utilizing intrapleural fibrinolysis therapy. This involved administering a total of three doses of a combination of intrapleural alteplase and DNase within a 24-hour timeframe. The pleural fluid drainage achieved with this approach proved to be highly effective, comparable to the outcomes observed in the Second Multicentre Intrapleural Sepsis Trial (MIST-2) [17].

Nosocomial outbreaks and secondary cases in close contacts are common; therefore, infection control measures and prophylactic antibiotics for high-risk contacts are crucial aspects of disease control. However, the lack of *S. pyogenes* vaccinations leaves our coverage of bacterial vaccines insufficient. Additionally, one of the limitations of our research is our inability to define the M protein serotype of the *S. pyogenes* strain discovered in our patient. Further study is needed to discover additional factors, such as previous viral infections other than influenza, and mechanisms for worsening *S. pyogenes* pneumonia.

## Conclusions

This case report highlights the unique presentation of *S. pyogenes* pneumonia in a patient with a history of asthma following influenza virus B infection. The rarity of *S. pyogenes* as a cause of community-acquired pneumonia underscores the importance of considering less common pathogens, especially in individuals with underlying respiratory conditions. The clinical course of this patient, marked by severe respiratory distress, pleural effusion, and prolonged mechanical ventilation, serves as a reminder of the potential severity of *S. pyogenes* pneumonia. Early recognition and initiation of appropriate antibiotic therapy are essential in managing such cases, and vaccination against influenza plays a critical role in reducing the risk of secondary bacterial infections. Healthcare providers should maintain a high index of suspicion for rare pathogens, even in cases of community-acquired pneumonia that do not fit the typical profile.
